# Maternal exercise increases infant resting energy expenditure: preliminary results

**DOI:** 10.1038/s41366-024-01560-0

**Published:** 2024-06-10

**Authors:** Filip Jevtovic, David N. Collier, James DeVente, Steven Mouro, Alex Claiborne, Breanna Wisseman, Dylan Steen, Kara Kern, Nicholas Broskey, Linda E. May

**Affiliations:** 1 Department of Kinesiology, East Carolina University, Greenville, NC 27858 USA; 2 Human Performance Laboratory, East Carolina University, Greenville, NC 27858 USA; 3 East Carolina Diabetes and Obesity Institute, East Carolina University, Greenville, NC 27834 USA; 4 Department of Pediatrics, Brody School of Medicine, East Carolina University, Greenville, NC 27834 USA; 5 Department of Obstetrics and Gynecology, Brody School of Medicine, East Carolina University, Greenville, NC 27834 USA

**Keywords:** Lifestyle modification, Nutrition, Paediatrics, Energy metabolism

## Abstract

Maternal obesity is associated with lower infant resting energy expenditure (REE), predisposing them to more rapid weight and adiposity gain through early infancy. Maternal exercise (ME) decreases infant adiposity and risk for childhood obesity; however, it remains unknown if this is in part mediated by changes in infant energy expenditure. Thus, we measured REE in 1-month-old infants from pregnant individuals who performed moderate-intensity exercise during pregnancy and compared it to infants from non-exercising controls. We observed higher oxygen respiratory rates (*p* = 0.003 for VO_2_ and *p* = 0.007 for VCO_2_) and REE (*p* = 0.002) in infants exposed to exercise in utero, independent of any differences in infant body composition. Furthermore, maternal BMI was significantly and inversely associated with infant REE in the control (*r* = −0.86, *R*^2^ = 0.74, *p* = 0.029), but not the exercise group (*r* = 0.33, *R*^2^ = 0.11, *p* = 0.473). Together, these findings associate ME with increasing infant energy expenditure which could be protective of subsequent infant adiposity gain. Clinical Trial: ClinicalTrials.gov Identifier: NCT03838146 and NCT04805502.

## Introduction

With rates of pediatric obesity escalating worldwide, it is crucial to investigate intervention and prevention strategies to help mitigate the propagation of obesity across generations. Apart from genetic and epigenetic predispositions, obesity development is dependent on energetic imbalance and caloric consumption that exceeds caloric expenditure. Accordingly, to mitigate obesity development, adjustment of caloric intake and/or caloric output is necessary. In adult populations, diet and exercise are factors that are often manipulated to control energy balance; however, in early infancy (<6 months of life), only dietary patterns can be adjusted as energetic output is primarily dependent on the infant’s resting energy expenditure due to a limited range of physical activity.

Maternal overweightness and obesity have been associated with lower infant energy expenditure within the first 6 months of life [1, 2], and associated with higher adiposity. Recently, we observed that maternal exercise (ME), independent of maternal BMI, increases infant umbilical cord-derived mesenchymal stem cell, a model for investigation of infant tissue metabolism, energy turnover and respiration [3]; however, if such effect is evident on a whole organism level remains unknown. To investigate this, we measured resting energy expenditure in infants exposed to moderate-intensity exercise in utero pregnancy. We hypothesized that infants exposed to exercise in utero will have higher resting energy expenditure, independent of any infant body composition differences.

## Methods

### Pre-intervention testing, exercise intervention, and maternal measurements

All methods except infant indirect calorimetry have been described elsewhere [3–5]. The study was approved by the East Carolina University Review Board and informed consent was obtained from each participant upon enrollment. Healthy females with various BMIs, between 18–40 years of age, with singleton pregnancy, without chronic disease, and use of any medication or substances (e.g., SSRI, tobacco) that could affect fetal development were recruited <16 weeks of gestation. Upon enrollment, participants underwent a submaximal modified Balke treadmill test [6] and were assigned their target heart rate (THR) zones that corresponded to maternal HR at 60–80% of maximal oxygen consumption (VO_2peak_). Two control participants enrolled during COVID-19 and THR was based on the self-reported pre-pregnancy physical activity level and age, using published guidelines [6]. After determining THR zones for each participant, women were randomly assigned (computer-generated randomization, GraphPad Prism Software) to aerobic, resistance, combination (aerobic + resistance) exercise, or a control group; however, for the current study all 3 exercise groups were combined into a single “(ME) group”. Upon randomization, participants exercised according to the American College of Obstetricians and Gynecologists guidelines for the duration of their pregnancy (~16–40 weeks). To ensure that participants performed moderate-intensity exercise (60–80% maximal oxygen consumption and 12–14 rated perceived exertion), every session was supervised, and heart rate was continuously monitored to ensure that participants stayed in their THR zones. The control group performed supervised stretching, breathing, and flexibility exercises at low intensity (<40% VO_2peak_). ME adherence was calculated by dividing the number of sessions attended by the total number of possible sessions within the participants’ gestational period. Maternal age, parity, pre-pregnancy weight and height and body mass index (BMI, kg/m^2^), gestational diabetes mellitus status (yes or no), gestational weight gain (GWG), length of gestation, mode of delivery, and breastfeeding status, were abstracted from various sources including pre-screening eligibility and postpartum questionnaires as well as maternal and neonatal electronic health records. At 16 weeks of gestation, we determined maternal BMI, waist-to-hip ratio (3D body scanner, or manually (Gulick tape)), and body fat percentage via skinfold technique and age-adjusted equations [7, 8].

### Infant body composition and indirect calorimetry

Birth measurements (i.e., weight) and infant sex were extracted from neonatal electronic health records. At 4–6 weeks of age, infant weight, length, BMI, and body fat (%), were measured by trained staff in our pediatric lab. Body fat was calculated using skinfold method [9]. To validate our skinfold measurement, we have obtained infant adiposity using DEXA scan on 8 infants in parallel with obtaining their adiposity using skinfold method. DEXA derived net fat percentage was significantly correlated (*r* = 0.99, *R*^2^ = 98, *p* < 0.001) with skinfold body fat percentage validating the use of skinfold method for 1-month adiposity measurement for the rest of the cohort. Infant respiration (volume of O_2_ and CO_2_) and REE were measured using TrueOne 2400 (PARVO Medics, Sandy, UT, USA) indirect calorimetry system [10, 11]. Indirect calorimetry assessment took place in a thermoneutral room, in low ambient lighting while infant rested, using an infant-specific hood canopy system across ~30 min, or until steady state respiration is reached/obtained. The initial 5–10 min of data was discarded to allow the measures to reach steady state and any infant movement was monitored and later excluded from REE analysis. Infants were “fasted” for a minimum of 1 h prior to the visit.

### Statistics

Unpaired parametric and nonparametric two-tailed t-tests with statistical significance set apriori at *p* < 0.05 were performed where appropriate using GraphPad Prism version 9.3 (GraphPad Software, San Diego, CA). Variance was similar between groups. Pearson correlations were performed to test any correlations between infant and maternal measurements. ANCOVAs were performed to test group differences while controlling for covariates (i.e., infant sex) using JMP Pro 17 (SAS, Cary, NC).

## Results

Maternal and infant characteristics are presented in Table 1. Twelve out of 13 pregnant individuals identified as Caucasian, and 1 mom identified as Black. Average adherence to exercise was 88.4 ± 7.1%. All pregnant individuals were free of gestational diabetes. Nine out of 13 pregnant individuals were over and 4/13 were within the National Academy of Medicine recommended gestational weight gain; however, distribution was similar between groups (control, 2/4; exercise, 2/5; *p* = 0.93). All infants were breastfed. Importantly, at 1 month of age, infants had similar body composition, including weight, length, BMI, abdominal circumference, body fat.

**Table 1:** Maternal and infant characteristics.

	Control (*n* = 6)	Exercise (*n* = 7)	p-value
Maternal characteristics			
Age	31.9 ± 3.2	29.9 ± 4.7	0.39
VO2peak (ml/kg/min) at enrollment^a^	22.0 ± 4.7	21.1 ± 6.9	0.83
Pre-pregnancy BMI	29.6 ± 6.2	29.3 ± 7.3	0.95
BMI at 16-weeks of gestation	30.6 ± 5.8	30.1 ± 7.5	0.91
Body Fat (%) at 16-weeks of gestation^a^	37.0 ± 4.3	35.2 ± 4.5	0.53
Waist-to-hip ratio at 16-weeks of gestation^a^	0.86 ± 0.1	0.87 ± 0.1	0.88
Gestational weight gain (kg)	19.6 ± 12.6	17.9 ± 14.0	0.83
Parity	1 (1, 2)	0 (0, 3)	0.63
Gestation length (weeks)	38.9 ± 0.9	39.5 ± 1.2	0.11
Mode of delivery (SVD/C-section)	3/3	4/3	0.99
Neonate characteristics			
Fetal sex (M/F)	2/4	4/3	0.59
Birth weight (kg)	3.5 ± 0.05	3.2 ± 0.04	0.47
Birth length (m)	0.48 ± 0.03	0.49 ± 0.02	0.41
Birth BMI	14.6 ± 2.2	13.1 ± 1.4	0.16
Weight (kg) at 1 month	0.43 ± 0.01	0.43 ± 0.01	0.89
Length (m) at 1 month	0.48 ± 0.03	0.49 ± 0.2	0.41
Infant BMI at 1 month	16.27 ± 1.7	15.0 ± 1.6	0.2
Head circumference (m) at 1 month	0.37 ± 0.02	0.34 ± 0.01	0.41
Abdominal circumference (m) at 1 month	0.38 ± 0.02	0.34 ± 0.01	0.27
Tricep+subscapular skinfold thickness (mm) at 1 month	13.4 ± 3.7	11.9 ± 4.2	0.51
Body fat (%) at 1 month	13.0 ± 3.7	11.4 ± 4.3	0.5
Apgar-1 min	8 (8, 9)	9 (8, 9)	0.27
Apgar-5 min	9 (9, 9)	9 (9, 9)	0.99

Normally distributed data—expressed as mean ± SD, non-parametric *t*-tests, *p* < 0.05; Data not normally distributed—expressed as median (min, max), Mann–Whitney test, *p* < 0.05.

^a^*n* = 1–2 data missing; measured at 13–16 weeks of gestation.

**Fig. 1:**
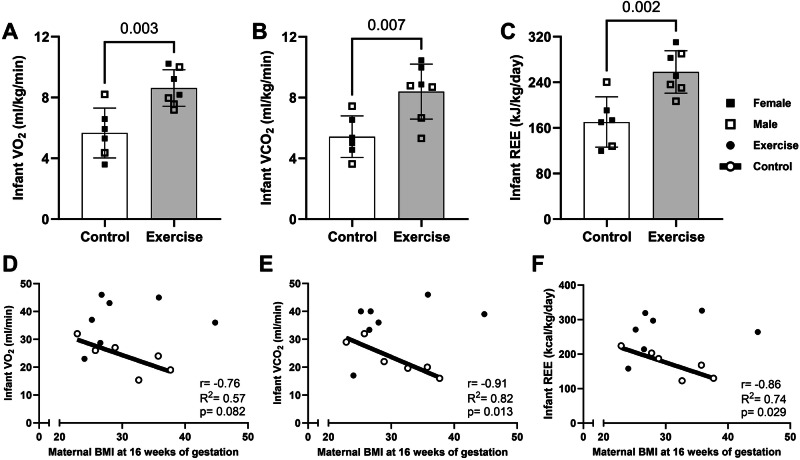
One-month Infant Resting Energy Expenditure relative to maternal exercise or BMI. Infants exposed to exercise in utero display higher rates of oxygen consumption (**A**), carbon dioxide expiration (**B**), and REE (**C**) normalized to body weight. This was seemingly void of any infant sex differences. Maternal BMI associations with infant respiratory measurements were only observed in the control group as seen with best fit regression line (**D**–**F**); however, this association is not present in the exercise group (black dots, regression line not shown). REE resting energy expenditure, VO_2_/VCO_2_ volume of oxygen and carbon dioxide, BMI body mass index. Data expressed as mean ± SD. *T*-test, Pearson correlation.

Infant VO_2_ (*p* = 0.01, *t* = 3.12), VCO_2_ (*p* = 0.015, *t* = 2.89), and REE (*p* = 0.009, *t* = 3.17) were significantly higher in exercise exposed compared to control group. When normalized to infant body weight, infants in exercise group had significantly higher VO_2_ (*p* = 0.003, *t* = 3.75) and VCO_2_ (*p* = 0.007, *t* = 3.28) which resulted in 87.9 kJ (21.0 kcal) higher daily energy expenditure (*p* = 0.002, *t* = 3.90) per kg of infant body weight (Fig. 1A–C). Of note, significant group differences remained when infant body weight was included as a covariate in ANCOVA and when the respiration values were divided by infant body weight as presented in Fig. 1, as well as when infant sex and/or race and/or mode of delivery were included as covariates in analysis. Infant respiratory exchange ratio (VCO_2_/VO_2_) was not significantly different between groups (*p* > 0.05). Finally, we found that maternal BMI at 16 weeks had a trend toward significant association with infant VO_2_, and significantly and inversely associated with infant VCO_2_ and REE (*r* = −0.76–−0.91, *R*^2^ = 0.57–0.82, *p* = 0.013–0.082; Fig. 1D–F) in the control group, but not exercise group (*p* > 0.05). Infant REE, VO_2_, or VCO_2_ were not significantly associated with maternal VO_2peak_.

## Discussion

To our knowledge, this is the first study to show that maternal moderate intensity exercise during pregnancy increases infant energy expenditure in early infancy, regardless of maternal BMI. It is important to note that higher infant energy expenditure was independent of differences in infant body composition. Specifically, higher respiration and energy expenditure in the exercise group persisted after normalizing to infant body weight (Fig. 1) and calculated (body weight − adipose tissue mass) lean mass (*p* = 0.003, *t* = 3.71; data not shown). These data suggest that factors beyond relative differences in body composition between groups contribute to the higher infant metabolic rate. In animal models ME increases brown adipogenesis and browning of white adipose tissue, increasing the adipose tissue mitochondrial uncoupling and offspring whole body respiration [12, 13]. Furthermore, ME drives futile calcium cycling to increase skeletal muscle-based thermogenesis [14]. While these effects have been corroborated in animal models, it remains to be elucidated if higher REE in infants is a factor of greater brown adipose tissue depot, browning of adipocytes, or provision of futile, ‘energy-wasting’ cycles (i.e., calcium cycling, lipid cycling, creatine cycling, etc.).

Maternal obesity has been associated with lower infant energy expenditure [1, 2], and we recapitulated that association within the control, but not exercise group (Fig. 1D–F). Such data suggests that exercise could be protective of maternal obesity-induced decrease in infant REE, potentially nullifying the previously established correlation. While this requires further investigation it is important to note that ME-induced increase in infant energy expenditure could be protective of subsequent adiposity gain, as lower energy expenditure was associated with a higher incidence of infants becoming overweight. Furthermore, such changes in REE with exercise (or maternal obesity) could contribute to suboptimal nutritional prescription, particularly as infant formula quantity (and other feeding practices) is predominantly based on the infant’s age and body weight. Together, although it is hard to determine the energetic needs of an infant within the clinic, environmental factors experienced by pregnant individuals that could influence intrauterine fetal development should be considered when determining infant energetic needs.

In conclusion, this preliminary data from our randomized control trial showcases that maternal moderate-intensity exercise during pregnancy is associated with an increase in infant respiration and energy expenditure independent of body composition differences. While our preliminary study includes a small subject population and lacks racial and ethnic diversity, our future studies will aim to address this.

## Data Availability

Data generated/analyzed during the current study are available upon request from the corresponding or lead author.
